# Splenectomy in Onco-Hematologic Patients: A Retrospective Study of Early Complications and 1-Year Mortality

**DOI:** 10.3390/cancers17132241

**Published:** 2025-07-04

**Authors:** Marion Faucher, Stanislas Ravot, Loïc Barthes, Jean Manuel de Guibert, Laurent Chow-Chine, Frédéric Gonzalez, Magali Bisbal, Luca Servan, Marie Tezier, Maxime Tourret, Sylvie Cambon, Camille Pouliquen, Damien Mallet, Lam Nguyen Duong, Florence Ettori, Jacques Ewald, Marc Léone, Antoine Sannini, Jonathan Garnier, Djamel Mokart

**Affiliations:** 1 Department of Anesthesiology and Critical Care, Paoli-Calmettes Institute, 13009 Marseille, France; faucherm@ipc.unicancer.fr (M.F.); stanislas.ravot@wanadoo.fr (S.R.); loic.barthes@laposte.net (L.B.); deguibertjm@ipc.unicancer.fr (J.M.d.G.); chowchinel@ipc.unicancer.fr (L.C.-C.); gonzalezf@ipc.unicancer.fr (F.G.); bisbalm@ipc.unicancer.fr (M.B.); servanl@ipc.unicancer.fr (L.S.); tezierm@ipc.unicancer.fr (M.T.); tourretm@ipc.unicancer.fr (M.T.); cambons@ipc.unicancer.fr (S.C.); pouliquenc@ipc.unicancer.fr (C.P.); malletd@ipc.unicancer.fr (D.M.); nguyenduongl@ipc.unicancer.fr (L.N.D.); ettorif@ipc.unicancer.fr (F.E.); sanninia@ipc.unicancer.fr (A.S.); 2 Department of Surgery, Paoli-Calmettes Institute, 13009 Marseille, France; ewaldj@ipc.unicancer.fr (J.E.); garnierj@ipc.unicancer.fr (J.G.); 3 Department of Anesthesiology and Critical Care, Hôpital Nord, 13009 Marseille, France; marc.leone@ap-hm.fr

**Keywords:** splenectomy, cancer, postoperative sepsis, mortality, postoperative complications

## Abstract

**Background:** Splenectomy remains necessary in selected oncologic and hematologic indications but is associated with significant postoperative morbidity and mortality. The data on outcomes in this high-risk population remain limited, particularly in mixed cohorts. **Methods**: We conducted a retrospective cohort study including all patients undergoing splenectomy for oncologic or hematologic causes between 2009 and 2022 at a cancer referral center. The primary outcomes were the occurrence of major complications at day 90 and the 1-year all-cause mortality. Multivariate logistic regression was used to identify independent predictors. **Results:** Among the 8503 ICU admissions from surgical wards, 204 splenectomies were performed; 179 patients were analyzed. The median age was 64 years, and 100 patients (55.9%) were female. Splenectomy was performed for hematologic malignancies in 76 cases (42.5%) and for oncologic causes in 103 cases (57.5%). Laparotomy was used in 154 cases (86.0%), and metastasectomy was performed in 54 patients (30.2%). At day 90, 86 patients (48.0%) developed a major complication: 12 deaths (6.7%), 44 surgical complications (24.6%), and 71 episodes of sepsis (39.7%). In a multivariate analysis, weight loss (OR 3.39, 95% CI [1.32–8.70], *p* = 0.011), laparotomy (OR 4.38 [1.09–17.60], *p* = 0.038), and a higher SAPS II score (OR 1.08 per point [1.03–1.13], *p* = 0.003) were associated with complications, while metastasectomy was protective (OR 0.23 [0.08–0.67], *p* = 0.007). At one year, the mortality reached 22.4%. Independent predictors of death were sepsis at one year (OR 5.04, 95% CI [1.30–25.96], *p* = 0.029), the Charlson Comorbidity Index (OR 1.30 per point, 95% CI [1.04–1.68], *p* = 0.030), invasive mechanical ventilation (OR 14.94, 95% CI [2.83–118.93], *p* = 0.003), and a performance status >1 (OR 7.84, 95% CI [2.38–27.75], *p* < 0.001). Encapsulated bacteria were not isolated; sepsis was mainly due to Gram-negative and enterococcal organisms. **Conclusions**: Splenectomy in onco-hematologic patients is associated with high rates of sepsis and mortality. In addition to surgical factors, frailty, immune status, and infection independently contribute to the patients’ outcomes. These results support risk-adapted perioperative strategies and long-term infectious surveillance in immunocompromised patients.

## Simple Summary

The spleen helps protect the body from infections, especially those caused by encapsulated germs like *Streptococcus pneumoniae*. In some cancer patients, the spleen must be surgically removed—a procedure called splenectomy. We studied 179 patients with blood cancers or solid tumors who had undergone this operation. Almost half of them experienced serious complications within three months, and over 20% died within one year. Many patients developed infections or required invasive mechanical ventilation. The risk of complications was higher in the patients who had lost weight before surgery, were in poor general condition, or underwent open (rather than minimally invasive) surgery. Interestingly, patients whose spleen was removed as part of a planned cancer surgery to remove isolated metastases had fewer problems. Although the patients received vaccines and antibiotics, infections still occurred—mostly caused by hospital bacteria, not encapsulated germs. These findings show that splenectomy remains a high-risk procedure in cancer patients, especially those with blood diseases. Improving pre-surgery health, preventing infections, and providing close monitoring may help reduce complications.

## 1. Introduction

The spleen plays a central role in host immunity and hematologic homeostasis. Indeed, it contributes to both innate and adaptive immune responses by filtering bloodborne pathogens, removing opsonized bacteria—particularly encapsulated organisms—and serving as a reservoir for memory B cells and marginal zone macrophages. Its removal disrupts this immune surveillance, which may explain the increased incidence of severe infections and sepsis observed in certain splenectomized patients, especially those with underlying hematologic malignancies [1,2].

Although splenectomy has become less frequent in the era of targeted therapies and minimally invasive surgery, it remains necessary in selected oncologic and hematologic indications, including diagnostic, therapeutic, or hemostatic purposes [3]. However, in immunocompromised patients—particularly those with cancer—the procedure carries a significant burden of postoperative morbidity and long-term mortality [4]. Postoperative complications are a major concern in surgical oncology. Among them, sepsis is particularly feared, not only for its short-term severity but also for its impact on long-term outcomes, including survival and the oncologic trajectory. Postoperative infections may delay adjuvant treatments, impair immune surveillance, and potentially contribute to disease recurrence or death, although supporting evidence remains limited [5]. In hematologic malignancies, this risk is amplified by cytopenias, prior chemotherapy, and profound immune dysfunction [6]. Although overwhelming post-splenectomy infection (OPSI) has become rare thanks to antibiotic prophylaxis and vaccination, severe infections remain common and often involve hospital-acquired or opportunistic organisms, reflecting a more complex form of immunosuppression [7,8]. Beyond infection, other perioperative parameters such as frailty, comorbidities, and surgical complexity also modulate the patients’ outcomes [9]. However, their assessment remains underexplored in splenectomy cohorts, especially in populations that include both solid tumor and hematologic malignancies [10,11]. Few studies have examined the interplay between early postoperative events and 1-year mortality in this population [4,12,13]. Whether short-term complications such as sepsis or respiratory failure are isolated events or indicators of a longer-term prognosis remains an open question. Moreover, the differential outcomes between patients with solid tumors and those with hematologic malignancies undergoing splenectomy have yet to be clearly described. In this context, we conducted a retrospective cohort study of patients undergoing splenectomy for oncologic and hematologic indications at a comprehensive cancer center. Our aim was to describe the early and late postoperative outcomes and to identify the risk factors—particularly infectious and functional—that predict major complications at 90 days and mortality at one year.

## 2. Materials and Methods 

### 2.1. Study Design

We conducted a retrospective observational study at the Institut Paoli-Calmettes, a comprehensive cancer center in Marseille, France. All adult patients undergoing total splenectomy for solid tumors or hematologic indications, whether elective or emergency, between 9 February 2009 and 17 June 2022, were included. The study was approved by the Institutional Review Board (Splenecthor 2023-012) and conducted in accordance with the principles of the Declaration of Helsinki. In our institution, all patients scheduled for splenectomy are admitted to the intermediate care unit or the intensive care unit (IMC/ICU) for standard monitoring and postoperative care for a minimum of 2 days before their transfer to the surgical ward, except in the event of complications.

### 2.2. Inclusion and Exclusion Criteria

The eligible patients were ≥18 years of age and had undergone total splenectomy for an oncologic or hematologic indication, whether curative, diagnostic, or hemostatic. All patients admitted postoperatively to either the intensive care unit (ICU) or the intermediate care unit (IMC) were included. Patients undergoing partial splenectomy or splenectomy for non-oncologic, non-hematologic indications were excluded. Cases managed exclusively in conventional surgical wards could not be reliably captured and were therefore not included.

### 2.3. Data Collection

The clinical data were extracted from the electronic medical records using the institutional hospital and IMC/ICU information systems. All relevant preoperative, intraoperative, and postoperative information was collected through a review of surgical reports, anesthesia files, IMC/ICU records, and multidisciplinary tumor board decisions.

The preoperative variables included demographic data (age, sex), body mass index (BMI), weight loss of more than 3 kg during the 6 months before the intervention, preexisting comorbidities (including hypertension, diabetes, chronic respiratory or renal disease), previous corticosteroid or chemotherapy exposure, and preoperative hemoglobin level. The comorbidity burden was assessed using the Charlson Comorbidity Index (CCI) [4] and the American Society of Anesthesiologists (ASA) physical status. The functional status was documented through the Eastern Cooperative Oncology Group (ECOG) performance status (PS) and the metabolic equivalents of task (MET) [4]. We applied commonly used thresholds for risk stratification: an ASA > 2 to indicate moderate to severe systemic disease, an MET < 4 to reflect a limited exercise tolerance and increased perioperative risk, and an ECOG-PS > 1 to identify patients with functional impairment beyond minor symptoms. These cut-offs are frequently used in the perioperative period to define vulnerable populations. The presence of splenomegaly, a transfusion history within the month prior to surgery, and the vaccination status against encapsulated organisms was also recorded.

The intraoperative data included the surgical approach (laparotomy vs. laparoscopy), type of surgery (elective vs. emergency), indication (curative, diagnostic, metastasectomy), procedure duration, blood loss, transfusion requirement, and use of vasopressors. When splenectomy was performed for diagnostic purposes, the definitive indication was determined by a pathological examination. Cases involving metastasectomy were considered curative procedures when appropriate.

The postoperative data included the Simplified Acute Physiology Score (SAPS II) and the Sequential Organ Failure Assessment (SOFA) score (at admission and day 2), the IMC/ICU and hospital length of stay, and the need for organ support therapies, including vasopressors, oxygen > 5 L/min, high-flow nasal oxygen (HFNC), non-invasive ventilation (NIV), invasive mechanical ventilation (IMV), or renal replacement therapy (RRT). Infectious prophylaxis, antibiotic coverage, and thromboembolic prophylaxis or therapeutic anticoagulation were also recorded. Postoperative complications were classified as surgical or medical and graded according to the Clavien–Dindo classification. Sepsis was defined according to the Sepsis-2. When possible, microbiological documentation was obtained from blood cultures or site-specific sampling. Thromboembolic events (deep vein thrombosis, pulmonary embolism, arterial thrombosis, myocardial infarction, stroke) were identified up to one year postoperatively.

Two primary outcomes were evaluated. The first was the occurrence of major complications within 90 days after surgery. This composite outcome included any of the following: death, a Clavien–Dindo grade > II surgical complication, or the occurrence of one or more major organ failures requiring support (vasopressors, IMV, RRT, etc.). The second outcome was the all-cause mortality at one year following splenectomy, based on the medical records and national mortality registry data.

The patients were monitored for one year post-surgery using the hospital’s electronic system, which records all of the procedures, visits, laboratory tests, vital signs, and other relevant data, complete with dates and unique identifiers. The Paoli-Calmettes Institute adheres to a policy of regular follow-ups, scheduling at least one visit every three months following a major oncological surgery. In the cases of patients lost to follow-up, the INSEE database was employed to ascertain their deceased status (https://arbre.app/insee, last accessed on 15 February 2024).

### 2.4. Statistical Analysis

The data are presented as percentages for the qualitative variables and as medians [1st and 3rd quartiles] for the quantitative variables. The data were first compared between two patient groups: the presence or absence of a major complication within 90 days. The comparisons were made using the Mann–Whitney test for the continuous variables and the Chi-square or Fisher’s exact tests for the categorical variables. All *p*-values < 0.05 were considered statistically significant. Binary logistic regression analyses were used to identify the independent variables associated with major complications, measured by estimated odds ratios (ORs) and 95% confidence intervals (95% CIs). The variables with significance or borderline significance (*p* < 0.1) in the univariate analyses and those considered relevant from the literature were included in a multivariate regression model using stepwise backward selection. We chose 0.1 as the critical value for the model entry and exit. Statistical significance was set at *p* < 0.05. The Hosmer–Lemeshow test was used to verify the fit of the logistic model. The factors associated with 1-year mortality were analyzed using the same statistical method and R software, version 4.3. All tests were two-sided, and *p*-values < 0.05 were considered significant.

## 3. Results

**Figure 1:**
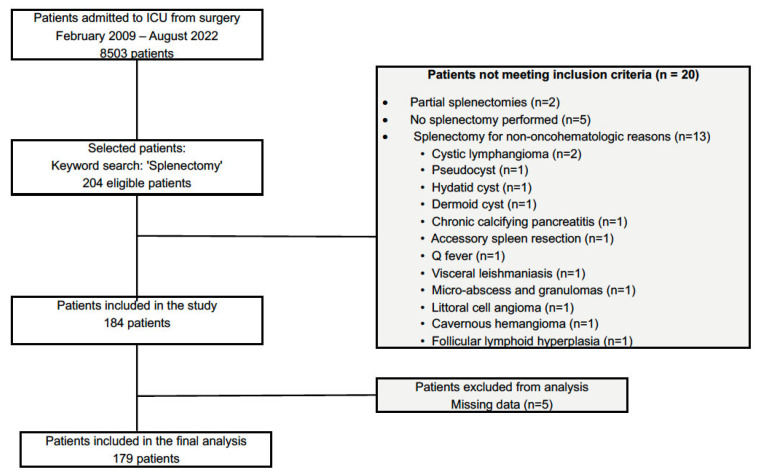
Patient flowchart for inclusion in the study cohort undergoing splenectomy for oncologic or hematologic malignancies (2009–2022).

Between 9 February 2009 and 17 June 2022, 8503 patients were admitted to the ICU from surgical wards, among whom 204 underwent splenectomy. Of these, 20 did not meet the inclusion criteria. A total of 184 patients were included in the study, and 5 were excluded due to missing data. Therefore, 179 patients were analyzed (Figure 1).

### 3.1. Patient Characteristics (Table 1)

The median age was 64 years [55.5–71.0], and 100 (55.9%) were female. A total of 62 patients (34.6%) had an ASA score > 2. Obesity (BMI > 30) was present in 25 patients (14.0%). Weight loss was reported in 69 cases (38.6%) and a performance status > 1 in 26 cases (14.5%). Preoperative anemia was common, with a median hemoglobin level of 11.85 g/dL [10.23–13.00].

**Table 1:** Baseline characteristics according to occurrence of major complications at day 90, with univariate analysis.

Patient Characteristics	Total Population (n = 179)	No Complication (n = 93)	Severe Complication (n = 86)	*p*-Value
Age (years)	64 [55–71]	63 [54–71]	65 [58–70]	0.400
Sex—Female	100 (55.9)	61 (65.6)	39 (45.3)	0.010
Sex—Male	79 (44.1)	32 (34.4)	47 (54.7)	
BMI (kg/m^2^)	23.9 [21.7–27.1]	23.6 [21.3–27.1]	23.9 [22.3–27.1]	0.284
Obesity (BMI > 30)	25 (14.0)	13 (14.0)	12 (14.0)	1.000
Weight loss	69 (38.6)	27 (29.0)	42 (48.8)	0.010
ASA score > 2	62 (34.6)	23 (24.7)	39 (45.3)	0.006
MET < 4	14 (7.8)	1 (1.1)	13 (15.1)	0.001
ECOG-PS > 1	26 (14.5)	7 (7.5)	19 (22.1)	0.011
Charlson Comorbidity Index	5 [3–7]	6 [4–8]	5 [3–7]	0.106
**Solid vs. Hematology Cancer**	103 (57.5)	57 (61.3)	46 (53.5)	0.366
Pancreas	31 (17.3)	12 (12.9)	19 (22.1)	0.154
Ovarian	24 (13.4)	17 (18.3)	7 (8.1)	0.077
Uterus	11 (6.1)	7 (7.5)	4 (4.7)	0.625
Gastric	11 (6.1)	6 (6.5)	5 (5.8)	1.000
Colon	7 (3.9)	4 (4.3)	3 (3.5)	1.000
Peritoneal disease	8 (4.5)	4 (4.3)	4 (4.7)	1.000
Other solid	15 (8.4)	7 (7.5)	8 (9.3)	0.874
Lymphomas	48 (26.8)	28 (30.1)	20 (23.3)	0.301
Leukemias	13 (7.3)	4 (4.3)	9 (10.5)	0.112
Myelofibrosis	17 (9.5)	5 (4.4)	12 (14.0)	0.051
**Preoperative Anticancer Treatments**				
Chemotherapy	109 (60.9)	58 (62.4)	51 (59.3)	0.790
Radiotherapy	15 (8.4)	9 (9.7)	6 (7.0)	0.703
Corticosteroids	25 (14.0)	7 (7.5)	18 (20.9)	0.018
Preoperative hemoglobin (g/dL)	11.8 [10.0–13.0]	12.0 [10.6–13.0]	11.0 [9.3–12.6]	0.010
Splenomegaly	96 (53.6)	44 (47.3)	52 (60.5)	0.107
Spleen length (cm)	13 [11–23]	12 [11–18]	14 [11–25]	0.051
Antibiotic prophylaxis	175 (97.8)	93 (100.0)	82 (95.3)	0.107
Penicillins	161 (90.0)	84 (90.3)	77 (95.1)	0.370
Others	14 (8.4)	10 (10.8)	4 (4.9)	0.260
Vaccination	165 (92.2)	92 (98.9)	73 (84.9)	0.012
**Intraoperative Period**				
Laparotomy	154 (86.0)	75 (80.6)	79 (91.9)	0.052
Laparoscopy	37 (20.7)	24 (25.8)	13 (15.1)	0.114
Elective surgery	162 (90.5)	93 (100.0)	69 (80.2)	<0.001
Emergency surgery	17 (9.5)	0 (0.0)	17 (19.8)	<0.001
Metastasectomy	54 (30.2)	39 (41.9)	15 (17.4)	0.001
**Surgical Indication**				
Invasion	42 (23.5)	19 (20.4)	23 (26.7)	0.388
Symptomatic splenomegaly	40 (22.4)	16 (17.2)	24 (27.9)	0.124
Thrombocytopenia	38 (21.2)	18 (19.4)	20 (23.3)	0.649
Diagnosis	30 (16.8)	13 (14.0)	17 (19.8)	0.403
Bleeding	18 (10.1)	5 (5.4)	13 (15.1)	0.052
Duration of surgery (hours)	3.5 [2.4–5.5]	3.4 [2.6–5.5]	4.0 [2.1–5.8]	0.932
Use of vasopressors	37 (20.7)	11 (11.8)	26 (30.2)	0.003
Blood loss (mL)	300 [150–675]	300 [100–500]	400 [200–850]	0.013

Results are expressed as numbers (percentages) for categorical variables and as medians [interquartile ranges] for continuous variables. BMI = body mass index; ASA = American Society of Anesthesiologists; MET = metabolic equivalent of task; ECOG-PS = Eastern Cooperative Oncology Group—performance status; SAPS II = Simplified Acute Physiology Score II; SOFA = Sequential Organ Failure Assessment.

### 3.2. Causal Disease Leading to Splenectomy (Table 1)

Surgery was performed for oncologic causes in 103 patients (57.5%) and for hematologic causes in 76 patients (42.5%). The median delay between the cancer diagnosis and splenectomy was 295 days [90–1232]. Splenomegaly was present in 96 cases (53.6%). Two patients had overlapping hematologic diagnoses (both leukemia and lymphoma), which explains the discrepancy between the total number of hematologic patients and the sum of the individual malignancy types.

### 3.3. Intraoperative Period (Table 1)

Laparotomy was the approach in 154 cases (86.0%), while laparoscopy was used in 25 cases (14.0%). Emergency surgery was performed in 17 cases (9.5%). Surgery was scheduled in 162 cases (90.5%) and performed for curative intent in 128 cases (71.5%). Metastasectomy was performed in 54 patients (30.2%). The median surgery duration was 3.5 h [2.39–5.50], and the median intraoperative blood loss was 300 mL [150–675]. Intraoperative vasopressors were used in 37 cases (20.7%), and transfusion was required in 42 patients (23.5%).

### 3.4. Postoperative Period (Table 2)

The median SOFA score at ICU admission was 2 [1–5], and the SAPS II score was 27 [21.25–35.00]. The ICU length of stay was 5 days [3–7], and the hospital length of stay was 11 days [8–16]. Surgical complications occurred in 68 patients (38.0%), of which 44 (24.6%) were Clavien–Dindo grade > 2. Reoperation was required in 23 patients (12.9%). Medical complications were reported in 96 cases (53.6%).

**Table 2:** Outcomes according to occurrence of major complications at day 90, with univariate analysis.

Outcomes	Total Population (n = 179)	No Complication (n = 93)	Severe Complication (n = 86)	*p*-Value
**Postoperative Period**				
SAPS II score	27 [21–35]	26 [21–30]	30 [23–39]	0.003
SOFA at ICU admission	2 [1–5]	1 [1–2]	3 [2–7]	<0.001
Surgical complications	68 (38.0)	21 (22.6)	47 (54.7)	<0.001
Clavien–Dindo grade > 2	44 (24.6)	0 (0.0)	44 (51.2)	<0.001
Reoperation	23 (12.9)	0 (0.0)	23 (26.7)	<0.001
Medical complications	96 (53.6)	29 (31.2)	67 (77.9)	<0.001
Sepsis at day 30	54 (30.2)	0 (0.0)	54 (62.8)	<0.001
Sepsis at day 90	71 (39.7)	0 (0.0)	71 (82.6)	<0.001
Thromboembolic event at Day 90	12 (6.7)	0 (0.0)	12 (15.6)	0.001
DVT	7 (3.9)	0 (0.0)	7 (9.1)	0.020
PE	4 (2.2)	0 (0.0)	4 (5.2)	0.086
Myocardial infarction	2 (1.1)	0 (0.0)	2 (2.6)	0.395
Stroke	1 (0.6)	0 (0.0)	1 (1.3)	0.924
Arterial thrombosis	1 (0.6)	0 (0.0)	1 (1.3)	0.924
Preventive anticoagulation	140 (78.2)	78 (90.7)	62 (84.9)	0.383
**Organ Failure at Day 90**				
Vasopressors	24 (13.4)	0 (0.0)	24 (27.9)	<0.001
Renal replacement therapy	8 (4.5)	0 (0.0)	8 (9.3)	0.015
O_2_ > 5 L/min	21 (11.7)	0 (0.0)	21 (24.4)	<0.001
High-flow oxygen	9 (5.0)	0 (0.0)	9 (10.5)	0.008
Non-invasive ventilation	11 (6.1)	0 (0.0)	11 (12.8)	0.001
Invasive mechanical ventilation	12 (6.7)	0 (0.0)	12 (14.0)	0.001
Time to severe complication (days)	2 [0–12]	NA	2 [0–12]	NA
IMC/ICU length of stay (days)	5 [3–7]	3 [0–5]	6 [3–8]	<0.001
Hospital length of stay (days)	11 [8–16]	9 [7–12]	16 [11–26]	<0.001
Death at day 90	12 (6.7)	0 (0.0)	12 (14.0)	0.001

Results are expressed as numbers (percentages) for categorical variables and as medians [interquartile ranges] for continuous variables. ICU = intensive care unit; IMC = intermediate care unit; DVT = deep vein thrombosis; PE = pulmonary embolism; O_2_ > 5 L/min = oxygen flow rate greater than 5 L per minute.

### 3.5. Prophylaxis and Supportive Care (Table 2)

Antibiotic prophylaxis was administered in 175 patients (97.8%), most commonly penicillin (161 patients, 90.0%). Vaccination against encapsulated organisms was documented in 165 patients (92.2%). Thromboprophylaxis was provided in 140 patients (78.2%).

### 3.6. Sepsis and Microbiological Documentation (Table 2)

Sepsis occurred in 54 patients (30.2%) within 30 days and in 94 patients (52.5%) within 1 year. Microbiological documentation was available in most cases. The main organisms isolated were *Escherichia coli*: 21 patients (11.7%), *Klebsiella pneumoniae*: 9 (5.0%), *Pseudomonas aeruginosa*: 7 (3.9%), *Enterobacter cloacae*: 5 (2.8%), *Enterococcus faecalis*: 5 (2.8%), and *Enterococcus faecium*: 5 (2.8%). No infections due to encapsulated organisms were identified. Notably, no encapsulated organisms such as *Streptococcus pneumoniae* or *Haemophilus influenzae* were isolated in any of the postoperative infections.

### 3.7. Organ Failures by Day 90 (Table 2)

Vasopressors were required in 24 patients (13.4%), RRT in 8 (4.5%), oxygen therapy > 5 L/min in 21 (11.7%), HFNC in 9 (5.0%), NIV in 11 (6.1%), and IMV in 12 (6.7%).

### 3.8. Thromboembolic Events (Table 2)

At day 90, thromboembolic events were observed in 12 patients (6.7%). At one year, 21 patients (11.7%) had experienced at least one thromboembolic event: deep vein thrombosis in 14 patients (7.8%), pulmonary embolism in 4 (2.2%), stroke in 1 (0.6%), myocardial infarction in 2 (1.1%), and arterial thrombosis in 1 (0.6%).

### 3.9. 30- and 90-Day Mortality (Table 2)

The mortality rate at 30 days was 8 cases (4.5%), and at 90 days, 12 patients (6.7%) had died.

### 3.10. Major Complications at Day 90 (Table 2)

At day 90, 86 patients (48.0%) had experienced at least one major complication. The median time to complication was 2 days [0–12]. This included 12 deaths (6.7%), 44 surgical complications classified as Clavien–Dindo grade > II (24.6%), and 23 reoperations (12.9%). Hemodynamic and respiratory support was frequently required: 13.4% needed vasopressors, 4.5% underwent renal replacement therapy, and 6.7% required invasive mechanical ventilation. Sepsis occurred in 71 patients (39.7%), and thromboembolic events were reported in 12 cases (6.7%). In the univariate analysis, several factors were significantly associated with major complications, including preoperative weight loss (48.8% vs. 29.0%, *p* = 0.010), an ASA score >2 (45.3% vs. 24.7%, *p* = 0.006), and an MET < 4 (15.1% vs. 1.1%, *p* = 0.001). Corticosteroid use, lower preoperative hemoglobin, hypertension, and intraoperative vasopressor use were also more frequent among patients with complications. Laparotomy was more common in this group (95.3% vs. 77.4%, *p* = 0.002), and SAPS II and SOFA scores were significantly higher postoperatively. Interestingly, metastasectomy was more frequent in the patients without complications (41.9% vs. 17.4%, *p* < 0.001). In the multivariate analysis, four variables remained independently associated with major complications at 90 days: weight loss (OR 3.39, 95% CI [1.32–8.70], *p* = 0.011), laparotomy (OR 4.38 [1.09–17.60], *p* = 0.038), a higher SAPS II score (OR 1.08 per point [1.03–1.13], *p* = 0.003), and metastasectomy, which remained a protective factor (OR 0.23 [0.08–0.67], *p* = 0.007).

### 3.11. 1-Year Mortality and Follow-Up (Table 3)

At one year, 40 patients (22.4%) had died. The median follow-up duration for the cohort was 31.1 months [12.67–67.90], with a total mortality rate of 54.8% at the last contact. No patients were lost to follow-up. The univariate analysis identified several clinical and perioperative variables significantly associated with 1-year mortality. An ASA score > 2 was present in 24 of 40 deceased patients (60.0%) compared to 38 of 139 survivors (27.3%, *p* < 0.001). A functional limitation (MET < 4) was recorded in 9 of 40 patients who died (22.5%) versus 5 of 139 survivors (3.6%, *p* < 0.001). Similarly, a performance status >1 was found in 13 deceased patients (32.5%) compared to 13 survivors (9.4%, *p* = 0.001). Comorbidities also played a major role: hypertension was observed in 24 of 40 patients who died (60.0%) versus 41 of 139 survivors (29.5%, *p* = 0.001), and diabetes in 13 patients (32.5%) versus 16 survivors (11.5%, *p* = 0.003). Corticosteroid exposure was more frequent among the deceased (25.0% vs. 10.8%, *p* = 0.043). The Charlson Comorbidity Index was significantly higher in the deceased patients (median 6.0 [4.0–8.0]) than in the survivors (median 5.0 [3.0–7.0], *p* = 0.002). The preoperative hemoglobin levels were significantly lower in the patients who died (median 10.45 g/dL) compared to the survivors (12.00 g/dL, *p* = 0.001). Regarding perioperative infectious prevention, suboptimal vaccination coverage (<100%) was more common in the mortality group: 30 of 40 patients (75.0%) versus 135 of 139 survivors (97.1%, *p* < 0.001). Sepsis within 30 days occurred in 21 of 40 deceased patients (52.5%) compared to 33 of 139 survivors (23.7%, *p* = 0.002). The need for vasopressors in the postoperative period was also more frequent in the mortality group (32.5% vs. 7.9%, *p* < 0.001), as was the requirement for invasive mechanical ventilation (IMV), which affected 10 of 40 deceased patients (25.0%) versus only 2 of 139 survivors (1.4%, *p* < 0.001). Among the 40 patients who died within 1 year of splenectomy, the disease status at the hospital discharge was stable in 27 patients (67.5%), in relapse in 4 (10%), in remission in 2 (5%), and progressing in 7 (17.5%). At 1 year, 23 patients (57.5%) were receiving palliative care. Among them, 12 were still receiving chemotherapy, while 11 had no ongoing treatment. Overall, 19 patients (47.5%) were on active chemotherapy at 1 year; of these, 7 were not in palliative care. Five additional patients were under surveillance with stable disease and no chemotherapy. For the remaining five patients, information on the care status at 1 year was unavailable. Notably, 30 of the 40 patients (75%) had an ECOG-performance status of 4 shortly before death, indicating severe functional deterioration.

**Figure 2:**
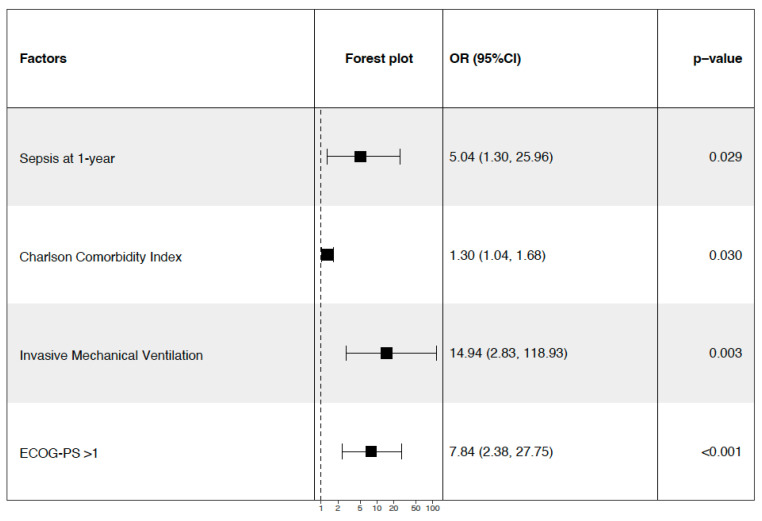
Forest plot of independent predictors of 1-year mortality following splenectomy. OR (95% CI): odds ratio with its 95% confidence interval.

In the multivariate analysis, four variables remained independently associated with 1-year mortality: sepsis at 1 year (OR 5.04, 95% CI [1.30–25.96], *p* = 0.029), the Charlson Comorbidity Index (OR 1.30 per point, 95% CI [1.04–1.68], *p* = 0.030), the use of invasive mechanical ventilation (OR 14.94, 95% CI [2.83–118.93], *p* = 0.003), and a performance status >1 (OR 7.84, 95% CI [2.38–27.75], *p* < 0.001) (Figure 2).

**Table 3:** Baseline characteristics according to 1-year vital status, with univariate analysis.

Patient Characteristics	Total Population (n = 179)	Alive at 1 Year (n = 139)	Death at 1 Year (n = 40)	*p*-Value
Age (years)	64 [55–71]	64 [55–70]	66 [59–73]	0.077
Sex—Female	100 (55.9)	80 (57.6)	20 (50.0)	0.505
Sex—Male	79 (44.1)	59 (42.4)	20 (50.0)	
BMI (kg/m^2^)	23.9 [21.7–27.1]	24.2 [21.9–27.4]	23.2 [21.1–24.3]	0.111
Obesity (BMI > 30)	25 (14)	19 (13.7)	6 (15)	1.000
Weight loss	69 (38.6)	49 (35.3)	20 (50.0)	0.132
ASA score > 2	62 (34.6)	38 (27.3)	24 (60.0)	<0.001
MET < 4	14 (7.8)	5 (3.6)	9 (22.5)	<0.001
ECOG-PS > 1	26 (14.5)	13 (9.4)	13 (32.5)	0.001
Charlson Comorbidity Index	5 [3–7]	5 [3–7]	6 [4–7]	0.093
**Solid vs. Hematology Cancer**	103 (57.5)	80 (57.6)	23 (57.5)	1.000
Pancreas	31 (17.3)	23 (16.5)	8 (20.0)	0.786
Ovarian	24 (13.4)	20 (14.4)	4 (10.0)	0.649
Uterus	11 (6.1)	10 (7.2)	1 (2.5)	0.474
Gastric	11 (6.1)	7 (5.0)	4 (10.0)	0.436
Colon	7 (3.9)	7 (5.0)	0	0.325
Peritoneal disease	8 (4.5)	7 (5.0)	1 (2.5)	0.803
Other solid	15 (8.4)	9 (6.5)	6 (15.0)	0.164
Lymphomas	48 (26.8)	37 (26.6)	11 (27.5)	0.903
Leukemias	13 (7.3)	9 (6.5)	4 (10)	0.681
Myelofibrosis	17 (9.5)	14 (10.1)	3 (7.5)	0.855
**Preoperative Anticancer Treatments**				
Chemotherapy	109 (60.9)	84 (60.4)	25 (62.5)	0.958
Radiotherapy	15 (8.4)	12 (8.6)	3 (7.5)	1.000
Corticosteroids	25 (14.0)	15 (10.8)	10 (25.0)	0.043
Preoperative hemoglobin (g/dL)	11.8 [10.2–13]	12 [10.5–13]	10.4 [9–12.2]	0.001
Splenomegaly	96 (53.6)	44 (47.3)	52 (60.5)	0.107
Spleen length (cm)	13 [11–23]	13 [11–21]	13 [11–25]	0.590
Antibiotic prophylaxis	175 (97.8)	139 (100.0)	36 (90.0)	0.001
Penicillins	161 (90.0)	129 (92.8)	32 (80.0)	1.000
Others	14 (8.4)	10 (7.2)	4 (10.0)	1.000
Vaccination	165 (92.2)	135 (97.1)	30 (75.0)	<0.001
**Intraoperative Period**				
Laparotomy	154 (86.0)	115 (82.7)	39 (97.5)	0.034
Laparoscopy	37 (20.7)	30 (21.6)	7 (17.5)	0.734
Elective surgery	162 (90.5)	131 (94.2)	31 (77.5)	0.004
Emergency surgery	17 (9.5)	8 (5.8)	9 (22.5)	0.004
Metastasectomy	54 (30.2)	49 (35.3)	5 (12.5)	0.010
**Surgical Indication**				
Invasion	42 (23.5)	29 (20.9)	13 (32.5)	0.159
Symptomatic splenomegaly	40 (22.4)	27 (19.4)	13 (32.5)	0.125
Thrombocytopenia	38 (21.2)	27 (19.4)	11 (27.5)	0.378
Diagnosis	30 (16.8)	25 (18.0)	5 (12.5)	0.563
Bleeding	18 (10.1)	10 (7.2)	8 (20.0)	0.033
Duration of surgery (hours)	3.5 [2.4–5.5]	3.5 [2.5–5.3]	4.2 [2.4–6.5]	0.143
Use of vasopressors	37 (20.7)	24 (17.3)	13 (32.5)	0.043
Bleeding (mL)	300 [150–675]	300 [125–500]	500 [225–950]	0.017
Intraoperative transfusion	42 (23.5)	24 (17.3)	18 (45.0)	<0.001
SAPS II score	27 [21–35]	26 [21–32]	34.5 [21–43]	0.032
SOFA score at ICU admission	2 [1–5]	2 [1–3]	5 [2–9]	0.001
Surgical complications	68 (38.0)	46 (33.1)	22 (55.0)	0.029
Clavien–Dindo grade > 2	44 (24.6)	25 (18.0)	19 (47.5)	<0.001
Reoperation	23 (12.9)	14 (10.1)	9 (22.5)	0.062
Medical complications	96 (53.6)	64 (46.0)	32 (80.0)	<0.001
Sepsis at 1 year	94 (52.5)	65 (46.8)	29 (72.5)	<0.001
Thromboembolic event at 1 year	21 (11.7)	17 (12.2)	4 (10.0)	0.085
DVT—n (%)	14 (7.8)	12 (8.6)	2 (5.0)	1.000
PE—n (%)	4 (2.2)	2 (1.4)	2 (5.0)	0.002
Myocardial infarction	4 (2.2)	3 (2.2)	1 (2.5)	0.468
Stroke	1 (0.6)	0 (0.0)	1 (2.5)	0.034
Arterial thrombosis	1 (0.6)	1 (0.7)	0 (0.0)	1.000
Preventive anticoagulation	140 (78.2)	112 (80.6)	28 (70.0)	0.900
**Organ Failure at Day 90**				
Vasopressors	24 (13.4)	11 (7.9)	13 (32.5)	<0.001
Renal replacement therapy	8 (4.5)	1 (0.7)	7 (17.5)	<0.001
Oxygen > 5 L/min	21 (11.7)	7 (5.0)	14 (35.0)	0.003
High-flow oxygen	9 (5.0)	5 (3.6)	4 (10.0)	0.786
Non-invasive ventilation	11 (6.1)	4 (2.9)	7 (17.5)	0.002
Invasive mechanical ventilation	12 (6.7)	2 (1.4)	10 (25.0)	<0.001
IMC/ICU length of stay (days)	5 [3–7]	4 [1.5–6]	7 [3–8]	0.001
Hospital length of stay (days)	11 [8–16]	1 [8– 14]	17 [10–26]	<0.001

Results are expressed as numbers (percentages) for categorical variables and as medians [interquartile ranges] for continuous variables. BMI = body mass index; ASA = American Society of Anesthesiologists; MET = metabolic equivalent of task; ECOG-PS = Eastern Cooperative Oncology Group—performance status; SAPS II = Simplified Acute Physiology Score II; SOFA = Sequential Organ Failure Assessment; ICU = intensive care unit; IMC = intermediate care unit; DVT = deep vein thrombosis; PE = pulmonary embolism; O_2_ > 5 L/min = oxygen flow rate greater than 5 L per minute.

### 3.12. Hematology Malignancy vs. Solid Tumor (Table 4)

Table 3 presents a comparison between the patients with hematologic malignancies (n = 76) and those with solid tumors (n = 103). The patients with hematologic cancers appeared more frail prior to surgery, with a higher proportion of an ASA score > 2 (47.4% vs. 25.2%, *p* = 0.004), an MET < 4 (14.5% vs. 2.9%, *p* = 0.010), and an ECOG-PS > 1 (25.0% vs. 6.8%, *p* = 0.001). These patients also had a higher rate of sepsis at day 90 (43.8% vs. 28.4%, *p* = 0.051) and at 1 year (66.7% vs. 44.3%, *p* = 0.006). Interestingly, the 90-day mortality was significantly higher in the hematologic group (11.8% vs. 2.9%, *p* = 0.040), while the 1-year mortality did not differ between the groups (22.4% vs. 22.3%, *p* = 1.000).

**Table 4:** Baseline characteristics according to cancer type (hematology vs. solid tumor cancers).

Patient Characteristics	Total Population (n = 179)	Hematology (n = 76)	Solid Tumors (n = 103)	*p*-Value
Age (years)	64 [55–71]	64 [56–70]	64 [55–71]	0.550
Sex—Female	100 (55.9)	37 (48.7)	63 (61.2)	0.131
Sex—Male	79 (44.1)	39 (51.3)	40 (38.8)	
BMI (kg/m^2^)	23.9 [21.7–27.1]	23.9 [21.5–26.7]	23.9 [21.8–27.8]	0.557
Obesity (BMI > 30)	25 (14.0)	3 (3.9)	22 (21.4)	0.002
Weight loss	69 (38.6)	23 (30.3)	46 (44.7)	0.072
ASA score > 2	62 (34.6)	36 (47.4)	26 (25.2)	0.004
MET < 4	14 (7.8)	11 (14.5)	3 (2.9)	0.010
ECOG-PS > 1	26 (14.5)	19 (25.0)	7 (6.8)	0.001
Charlson Comorbidity Index	5 [3–7]	24 [18–29]	11 [10–13]	<0.001
**Preoperative Anticancer Treatments**				
Chemotherapy	109 (60.9)	49 (64.5)	60 (58.3)	0.491
Radiotherapy	15 (8.4)	8 (10.5)	7 (6.8)	0.537
Corticosteroids	25 (14.0)	24 (31.6)	1 (1.0)	<0.001
Preoperative hemoglobin (g/dL)	11.8 [10–13.0]	10.4 [9.0–12.2]	12.2 [11–13.3]	<0.001
Splenomegaly	96 (53.6)	68 (89.5)	28 (27.2)	<0.001
Spleen length (cm)	13 [11–23]	12 [11–18]	14 [11–25]	0.051
Antibiotic prophylaxis	175 (97.8)	75 (98.7)	100 (97.1)	0.849
Penicillins	161 (90.0)	68 (94.4)	97 (97.0)	0.571
Others	14 (8.4)	8 (10.8)	6 (6.0)	0.383
Vaccination	165 (92.2)	92 (98.9)	73 (84.9)	0.656
**Intraoperative Period**				
Laparotomy	154 (86.0)	54 (71.1)	100 (97.1)	<0.001
Laparoscopy	37 (20.7)	29 (38.2)	8 (7.8)	<0.001
Elective surgery	162 (90.5)	69 (90.8)	92 (89.3)	0.943
Emergency surgery	17 (9.5)	6 (7.9)	11 (10.7)	0.711
Metastasectomy	54 (30.2)	7 (9.2)	47 (45.6)	<0.001
**Surgical Indication**				
Invasion	42 (23.5)	0 (0.0)	42 (40.8)	<0.001
Symptomatic splenomegaly	40 (22.4)	16 (17.2)	24 (27.9)	0.124
Thrombocytopenia	38 (21.2)	38 (50.0)	0 (0.0)	<0.001
Diagnosis	30 (16.8)	28 (36.8)	2 (1.9)	<0.001
Bleeding	18 (10.1)	4 (5.3)	14 (13.6)	0.120
Duration of surgery (hours)	3.5 [2.4–5.5]	2.5 [2.0–3.2]	5.0 [3.3–6.5]	<0.001
Use of vasopressors	37 (20.7)	4 (5.3)	33 (32.0)	<0.001
Blood loss (mL)	300 [150–675]	300 [100–800]	350 [162–575]	0.868
Intraoperative transfusion	42 (23.5)	23 (30.7)	19 (18.4)	0.086
**Postoperative Period**				
SAPS II score	27 [21–35]	29 [24–39]	26 [20–33]	0.081
SOFA at ICU admission	2 [1–5]	3 [2–5]	2 [1–3]	0.025
Severe complications	86 (48)	40 (52.6)	46 (44.7)	0.291
Surgical complications	68 (38.0)	25 (32.0)	43 (41.7)	0.242
Clavien–Dindo grade > 2	44 (24.6)	16 (21.1)	28 (27.2)	0.444
Reoperation	23 (12.9)	7 (9.3)	16 (15.5)	0.321
Medical complications	96 (53.6)	42 (54.7)	54 (52.4)	0.886
Sepsis at day 30	54 (30.2)	21 (28.0)	32 (31.1)	0.783
Sepsis at day 90	71 (39.7)	32 (43.8)	29 (28.4)	0.051
Sepsis at 1 year	91 (51%)	48 (66.7)	43 (44.3)	0.006
Thromboembolic event at day 90	12 (6.7)	5 (6.5)	7 (7.0)	1
DVT	7 (3.9)	4 (4.4)	3 (3.0)	0.952
PE	4 (2.2)	2 (2.9)	2 (2.0)	1.000
Myocardial infarction	2 (1.1)	0 (0.0)	2 (2.0)	0.654
Stroke	1 (0.6)	0 (0.0)	1 (1.0)	1.000
Arterial thrombosis	1 (0.6)	0 (0.0)	1 (1.0)	1.000
Thromboembolic event at 1 year	21 (11.7)	7 (9.8)	14 (16.7)	0.350
Preventive anticoagulation	140 (78.2)	58 (95.1)	82 (83.7)	0.057
**Organ Failure at Day 90**				
Vasopressors	24 (13.4)	7 (9.2)	17 (16.5)	0.095
Renal replacement therapy	8 (4.5)	2 (2.7)	5 (4.9)	0.726
O2 > 5 L/min	21 (11.7)	7 (9.2)	14 (13.6)	0.506
High-flow oxygen	9 (5.0)	5 (6.5)	4 (3.9)	0.940
Non-invasive ventilation	11 (6.1)	3 (4.0)	8 (7.8)	0.474
Invasive mechanical ventilation	12 (6.7)	4 (4.4)	8 (7.8)	0.474
Time to severe complication (days)	2 [0–12]	2 [0–15]	2 [0–8]	0.980
IMC/ICU length of stay (days)	5 [3–7]	2 [0–4]	5 [3–7]	<0.001
Hospital length of stay (days)	11 [8–16]	9 [6–12]	14 [9–18]	<0.001
Death at day 90	12 (6.7)	9 (11.8)	3 (2.9)	0.040
Death at 1 year	40 (22.3)	17 (22.4)	23 (22.3)	1

Results are expressed as numbers (percentages) for categorical variables and medians [interquartile ranges] for continuous variables. BMI = body mass index; ASA = American Society of Anesthesiologists; MET = metabolic equivalent of task; ECOG-PS = Eastern Cooperative Oncology Group—performance status; SAPS II = Simplified Acute Physiology Score II; SOFA = Sequential Organ Failure Assessment; ICU = intensive care unit; IMC = intermediate care unit; DVT = deep vein thrombosis; PE = pulmonary embolism; O_2_ > 5 L/min = oxygen flow rate greater than 5 L per minute.

## 4. Discussion

In this retrospective cohort of 179 patients undergoing splenectomy in an onco-hematologic setting, nearly half developed a major complication by day 90, and more than one-fifth died within one year. The multivariate analyses identified four independent predictors of 90-day complications and four of 1-year mortality, each of which reveals clinically meaningful insights.

### 4.1. Nutritional Status and Prehabilitation

Weight loss was strongly associated with short-term complications, likely reflecting preoperative malnutrition and frailty [14]. Nutritional status is known to correlate with adverse surgical outcomes, particularly in oncologic and hematologic patients [15,16]. Indeed, malnourished individuals demonstrate impaired wound healing, reduced immune competence, and diminished physiological reserve [17]. These findings are consistent with a recent study that emphasized the importance of preoperative nutritional status in elderly cancer patients undergoing major abdominal surgery [9]. Although direct evidence is scarce in this specific population, adapting selected ERAS principles—such as nutritional screening and prehabilitation—might help optimize the outcomes in oncologic and hematologic patients undergoing splenectomy. Similarly, structured postoperative strategies inspired by ICU bundles like ABCDEF could hold potential benefits for these often frail and immunocompromised individuals and deserve further investigation. These patients, often frail and immunocompromised, may particularly benefit from a structured, anticipatory approach [18,19].

### 4.2. Surgical Approach and Disease Context

An open surgical approach was independently associated with postoperative complications. This finding is in line with previous studies reporting increased morbidity with laparotomy compared to minimally invasive surgery, particularly regarding infection, bleeding, and prolonged recovery [20]. In our cohort, the high rate of laparotomy likely reflects the technical challenges posed by massive splenomegaly, hemorrhagic risk, or advanced disease—especially in hematologic malignancies, where minimally invasive access is often not feasible. This pattern reinforces prior observations in hematologic patients undergoing urgent abdominal surgery, where open procedures are frequently necessitated by the disease complexity and anatomical constraints [6]. Indeed, the high proportion of open splenectomies observed in our cohort reflects both clinical and technical considerations [21]. Our hematologic patients were frequently frail and affected by splenomegaly, which, while the primary indication in 22.4% of cases, was present in over half of the patients (53.6%). Moreover, splenectomy was often performed as part of complex surgical procedures—such as duodenopancreatectomy—in which laparoscopic approaches are not routinely used at our institution due to their limited applicability and technical complexity. In contrast, metastasectomy was associated with a lower risk of complications. All splenectomies performed in this setting were part of an oncologic strategy targeting isolated or oligometastatic disease, in patients with solid tumors, and notably without peritoneal carcinomatosis. These conditions likely contribute to favorable surgical and physiological profiles: the elective surgery timing, controlled tumor burden, limited need for extended cytoreduction, and better-preserved immune function. This contrasts sharply with the findings from ovarian cancer surgery, where splenectomy frequently signals a high tumor burden and an upper abdominal extension, and it is often performed within the context of extensive cytoreduction. As demonstrated in the study by Karlsson et al., splenectomy in ovarian cancer patients—particularly when associated with a high Peritoneal Cancer Index (PCI)—is a marker of high surgical complexity and is independently associated with increased postoperative complications and reduced overall survival rates [22]. In our cohort, the absence of carcinomatosis and the elective nature of metastasectomy suggest that splenectomy, in this context, is not a surrogate for aggressive disease but rather a reflection of localized, surgically manageable pathology. This may explain the paradoxical finding of a protective association between metastasectomy and complications and supports the idea that the tumor biology and surgical context should be jointly considered when interpreting the prognostic implications of splenectomy.

### 4.3. Immediate Postoperative Severity

We found that the effective and early management of organ failure, from the operating room admission to the first postoperative day, as evaluated using the SAPS II score, was critical in reducing the postoperative severe complications. Indeed, the SAPS II score was associated with early complications, reflecting the burden of acute illness at the IMC/ICU admission. It parallels the SOFA score in many ways, as both capture systemic dysfunctions that portend worse outcomes. Prior studies have shown that SAPS II and SOFA scores are independently associated with postoperative outcomes in onco-surgical patients [4,9,12]. Our observed 90-day mortality (6.7%) and complication (48%) rates are consistent with the published data on splenectomy in oncologic and hematologic populations, which report complication rates ranging from 17% to over 60% and mortality rates between 1.1% and 9%, depending on the disease severity and surgical context [23,24]. Importantly, all of the patients in our cohort were admitted postoperatively to an intermediate care unit, where enhanced monitoring allows for the early recognition and diagnosis of complications. This systematic high-acuity follow-up likely increases the detection rates compared to conventional surgical wards and may contribute to a higher reported incidence of complications—an observation we have previously documented [4]. Although sepsis could not be independently analyzed as a risk factor for severe complications—since it was included in their definition—it represented 39.7% of all severe events at day 90. This finding underscores the critical role of infectious complications in early postoperative morbidity and further justifies the emphasis on infection surveillance and prevention strategies in this high-risk population.

### 4.4. Immunity, Sepsis, and Long-Term Mortality

Sepsis within the first year was the strongest predictor of 1-year mortality. Despite excellent prophylactics, over 50% of the patients developed sepsis at one year, although no OPSI was documented. This highlights the effectiveness of vaccination and the lack of encapsulated organisms, in line with the literature reviews on post-splenectomy sepsis prevention [25]. However, the pathogens involved—mostly Gram-negative bacilli and enterococci—reflect a deeper immunodeficiency typical of cancer patients, combining postoperative asplenia and chemotherapy-induced immunosuppression [2,6,26]. In a recent study, postoperative sepsis was significantly associated with long-term mortality and cancer recurrence [12]. These findings echo earlier work showing that post-aggressive immunosuppression may foster tumor progression [4]. Along this line, IMV also emerged as a strong predictor of mortality. It often reflects severe sepsis, pulmonary complications, or multiorgan failure. In cancer patients, IMV is frequently needed for acute respiratory failure caused by infection, alveolar hemorrhage, or leukemic infiltration [27]. The association between postoperative IMV and sepsis supports a model of early infectious decompensation driving downstream mortality [4,12]. These findings advocate for intensive early monitoring and aggressive infection control strategies. As part of a sensitivity analysis, we compared the outcomes between patients with hematologic malignancies and those with solid tumors. The patients with hematologic cancers exhibited significantly more preoperative frailty and immunosuppression, and this translated into a higher incidence of sepsis at both day 90 and 1 year. These infections were associated with a significantly higher 90-day mortality, although the 1-year mortality did not differ between the groups. These findings suggest that early postoperative sepsis may disproportionately impact short-term outcomes in hematologic patients. This highlights the need for differentiated perioperative strategies, including optimized infection surveillance and possibly prehabilitation, tailored to the specific vulnerabilities of each oncologic subgroup. Finally, the indications for splenectomy in hematologic malignancies must be interpreted in light of the inclusion period of our study (2009–2022), during which the therapeutic options evolved substantially. At the beginning of the study period, splenectomy was more commonly used for diagnostic clarification, symptomatic splenomegaly, or refractory thrombocytopenia, particularly in conditions such as myelofibrosis or refractory thrombocytopenia. However, over time, the emergence of targeted therapies, including rituximab, ruxolitinib, and thrombopoietin receptor agonists, has progressively replaced surgery in many of these contexts [23,28,29,30]. Similarly, in indolent lymphomas or myeloproliferative neoplasms, effective systemic therapies can now reduce the splenic size and symptoms without surgical risk [31]. Our findings, particularly the higher 90-day mortality in hematologic patients, support a cautious, individualized approach, ideally involving a multidisciplinary discussion to reassess the benefit–risk balance of splenectomy in an era of expanding non-surgical options [32].

### 4.5. Comorbidity Burden and Performance Status

The CCI and the baseline ECOG-PS were both independently associated with 1-year mortality. These factors have practical implications, as they are assessable preoperatively and amenable to intervention [33,34]. Comorbidities and a poor ECOG-PS decrease physiological reserves, compromise tolerance to infections or chemotherapy, and are well-known predictors of poor survival in both oncologic and hematologic populations. The overlap between the CCI and the ECOG-PS argues for a unified approach to the preoperative assessment, combining geriatric, functional, and medical screenings. Both the CCI and the PS suggest a shared actionable axis: identifying vulnerable patients who may benefit from prehabilitation, comorbidity optimization, and targeted perioperative planning.

### 4.6. Limitations

This study has several limitations. First, its retrospective and observational design inherently carries a risk of selection bias. The patient inclusion was dependent on the availability and completeness of medical records, which may have excluded the most complex or undocumented cases. Moreover, unmeasured confounding factors may have influenced both the treatment decisions and the outcomes, despite the multivariate adjustments. As with all retrospective analyses, causal inferences should be made with caution. Finally, we did not use competing risk models such as the Fine–Gray model due to the absence of precise timing for postoperative complications in our dataset; however, the low 90-day mortality rate (6.7%) likely limited the impact of this limitation.

## 5. Conclusions

Our study offers a comprehensive perspective on splenectomy outcomes across both solid tumor and hematologic malignancy patients. The protective effect of metastasectomy underscores the generally more favorable surgical context of oncology patients—elective procedures, preserved immunity, and a lower perioperative risk. In contrast, hematologic patients remain highly vulnerable, with a sustained burden of infectious and respiratory complications despite optimal prophylaxis. By integrating the surgical parameters, immunological status, and validated severity scores, we provide a structured framework for perioperative risk stratification. These findings highlight the need for tailored pathways in hematology, where cytopenia, immunosuppression, and functional frailty converge to amplify the postoperative risk. Ultimately, the study advocates for a multidisciplinary strategy that includes immune profiling, comorbidity management, and prehabilitation—particularly in immunocompromised hosts. Incorporating such measures into the routine practice and institutional pathways may help reduce the preventable complications and improve the outcomes in this complex and diverse patient population.

## Data Availability

Data cannot be shared publicly because consent for publication of raw data was not obtained from study participants. Data are available from the Internal Review Board (IRB) of Institut Paoli Calmettes (contact via UTSI@ipc.unicancer.fr) for researchers who meet the criteria for access to confidential data.
